# A new kymogram-based method reveals unexpected effects of marker protein expression and spatial anisotropy of cytoskeletal dynamics in plant cell cortex

**DOI:** 10.1186/s13007-017-0171-9

**Published:** 2017-03-27

**Authors:** Fatima Cvrčková, Denisa Oulehlová

**Affiliations:** 1 Department of Experimental Plant Biology, Faculty of Sciences, Charles University in Prague, Prague, Czech Republic; 2 Institute of Experimental Botany, Academy of Sciences of the Czech Republic, Prague, Czech Republic

**Keywords:** Actin, Microtubules, Lifeact, Variable angle fluorescence microscopy, Spinning disc confocal microscopy, Kymogram, Structure stability, Lateral mobility, Anisotropy, FH1 (At3g25500)

## Abstract

**Background:**

Cytoskeleton can be observed in live plant cells in situ with high spatial and temporal resolution using a combination of specific fluorescent protein tag expression and advanced microscopy methods such as spinning disc confocal microscopy (SDCM) or variable angle epifluorescence microscopy (VAEM). Existing methods for quantifying cytoskeletal dynamics are often either based on laborious manual structure tracking, or depend on costly commercial software. Current automated methods also do not readily allow separate measurements of structure lifetime, lateral mobility, and spatial anisotropy of these parameters.

**Results:**

We developed a new freeware-based, operational system-independent semi-manual technique for analyzing VAEM or SDCM data, QuACK (Quantitative Analysis of Cytoskeletal Kymograms), and validated it on data from *Arabidopsis thaliana fh1* formin mutants, previously shown by conventional methods to exhibit altered actin and microtubule dynamics compared to the wild type. Besides of confirming the published mutant phenotype, QuACK was used to characterize surprising differential effects of various fluorescent protein tags fused to the Lifeact actin probe on actin dynamics in *A.* *thaliana* cotyledon epidermis. In particular, Lifeact-YFP slowed down actin dynamics compared to Lifeact-GFP at marker expression levels causing no macroscopically noticeable phenotypic alterations, although the two fluorophores are nearly identical. We could also demonstrate the expected, but previously undocumented, anisotropy of cytoskeletal dynamics in elongated epidermal cells of *A.* *thaliana* petioles and hypocotyls.

**Conclusions:**

Our new method for evaluating plant cytoskeletal dynamics has several advantages over existing techniques. It is intuitive, rapid compared to fully manual approaches, based on the free ImageJ software (including macros we provide here for download), and allows measurement of multiple parameters. Our approach was already used to document unexpected differences in actin mobility in transgenic *A.* *thaliana* expressing Lifeact fusion proteins with different fluorophores, highlighting the need for cautious interpretation of experimental results, as well as to reveal hitherto uncharacterized anisotropy of cytoskeletal mobility in elongated plant cells.

**Electronic supplementary material:**

The online version of this article (doi:10.1186/s13007-017-0171-9) contains supplementary material, which is available to authorized users.

## Background

Advanced microscopy methods such as spinning disc confocal microscopy (SDCM) or variable angle epifluorescence microscopy (VAEM) are nowadays becoming available to a wide community of researchers [1–4]. At the same time, new fluorescent protein markers are being developed, enabling “minimally invasive” detection of intracellular structures in vivo in practically any type of cells. Both main cytoskeletal systems—microtubules and microfilaments—are nowadays being routinely visualized in living cells of plant epidermis in situ using fluorescent protein marker expression in stable transgenic plants or transient transformants. Effects of biologically relevant conditions such as e.g. developmental changes, external influences or mutations on cytoskeletal organization and dynamics can be thus studied, leading to better understanding of the cellular mechanisms underlying plant ontogeny and responses towards environmental challenges.

Multiple markers for labeling either cytoskeletal system have been employed in plant studies. Widely used are fusion proteins based on fimbrin [5–7], albeit they can affect actin-dependent processes including organelle motility [8] and cause actin bundling when overexpressed [9]. At present, a 17 amino acid peptide called Lifeact is probably the least artifact-prone microfilament tag available, with negligible effects on in vitro actin polymerization kinetics over a wide concentration range [10], although its overexpression may stabilize microfilaments in vivo [11]. Expression levels and fluorophore properties can also influence actin structure and dynamics. For instance, red fluorescent proteins such as dsRed2 or mOrange either cause increased microfilament bundling or preferentially decorate existing bundles, while GFP and its derivatives fused to the same actin-binding moiety labeled finer filament networks. Overexpression of fimbrin derivatives tagged with mOrange in *Arabidopsis* seedlings caused growth inhibition, and even mild expression of a Lifeact-based marker slightly reduced root growth [9].

Several markers are available also for in vivo microtubule visualization [12, 13], and their use is plagued by similar problems. Frequently used are derivatives of the microtubule-associated protein MAP4 [14–16], which, however, induce organ twisting in *Arabidopsis* at high expression levels (e.g. [17]), indicating interference with cytoskeletal structure and dynamics, with subsequent effects on growth. Low-level expression of fluorescent protein-tagged tubulin subunits [18–20] can be used to label microtubules, since fluorescent protein-tagged tubulin, unlike tagged actin, can co-polymerize with native monomers. However, studies in a non-plant system (*Dictyostelium*) suggest that labeled tubulin may affect microtubule dynamics [21].

Only major changes to actin or microtubule organization and dynamics are obvious upon visual examination of microscopic images or video sequences. Additional insight can be obtained by inspection of kymograms—two-dimensional projections of the video sequence produced by plotting in the horizontal dimension pixels chosen from individual frames along a straight (linear) or curved transect, while the temporal dimension generates the vertical axis. Kymograms are sometimes used to qualitatively demonstrate plant cytoskeletal dynamics (e.g. [22–26]).

Quantitative image analysis is usually required to extract biologically relevant information from images and video sequences produced by advanced microscopy methods. Besides proprietary software (distributed often by the manufacturers of the microscopy equipment), freeware and shareware programs are being developed by the scientific community. In particular, ImageJ and its Fiji distribution [27] has recently become a standard part of the biologists´ toolkit, including utilities for generating kymograms.

For evaluation of plant cytoskeletal structure (i.e. static distribution of filaments), a number of particularly ImageJ-based protocols has been employed (e.g. [28–33]). Nevertheless, quantitative analysis of cytoskeletal dynamics remains challenging, especially in the case of rapidly changing and often weakly labeled actin structures, and techniques involving manual tracking or counting of individual filaments or filament bundles thus remain the method of choice (e.g. [23, 25, 26, 33, 34]). Such a manual approach is usually very laborious and may be prone to observer bias (which can be eliminated by a blinded study design involving an evaluator who does not know which sample is which). Development of partially or fully automated methods is thus desirable. To our knowledge, only one such technique—image-to-image correlation analysis—has found wider application in studies of plant cortical cytoskeleton dynamics [35, 36]. While this method produces informative and reproducible results, it is far from intuitive from the point of view of mathematically often somewhat naive biologists. It also can only measure a single composite parameter that reflects overall structure mobility, namely the rate at which the correlation coefficient between two subsequent images departs from the value of 1, characterizing a static system. Another drawback of the image-to-image correlation method is its dependence on the costly commercial MATLAB software package, as no version for free data analysis packages such as SciLab is available to date. Recently published ImageJ-compatible or standalone tools that enable semi-automated or automated extraction of quantitative information from kymograms [37, 38] might provide an interesting high-throughput alternative to manual single filament tracking, but their suitability for plant cells remains to be tested.

Here we present a new semi-manual, intuitive method for quantitative analysis of SDCM or VAEM recordings of fluorescence protein-labeled plant cytoskeleton. Our method, QuACK (Quantitative Analysis of Cytoskeletal Kymograms), is based on Fiji and can provide separate measurements of actin filaments or microtubules lifetime and lateral mobility. QuACK has been developed and validated using image data from wild type *Arabidopsis thaliana* plants and mutants defective in the actin-nucleating protein FH1 (At3g25500), a member of the formin (FH2 protein) family. Mutants lacking FH1 were previously shown by traditional methods to exhibit increased bundling and stability of microfilaments, as well as enhanced turnover of microtubule ends and generally faster microtubule dynamics [25, 26]. Besides of reproducing these phenotypes, QuACK revealed surprising differences in the dynamics of microtubules in plants expressing Lifeact with various fluorescent protein tags. We also successfully applied QuACK to characterize the spatial anisotropy of actin and microtubule dynamics of fully differentiated elongated epidermal cells of *Arabidopsis* hypocotyls and petioles. The results suggest, to our knowledge for the first time, reduced dynamics of actin bundles parallel to the longitudinal cell axis and decreased lateral mobility of microtubules aligned with the predominant direction of the cortical microtubule array.

## Results

### Method design

**Fig. 1:**
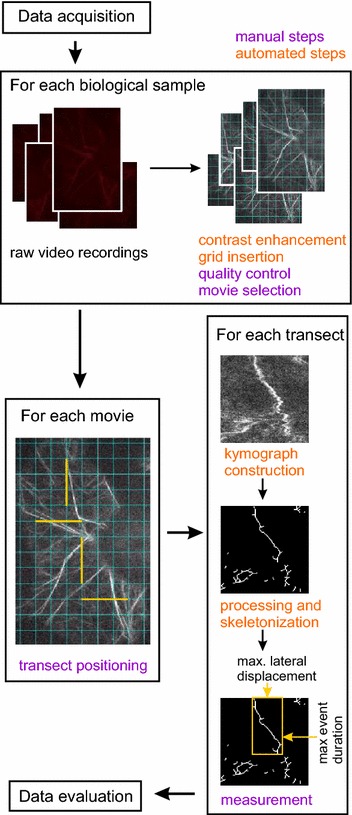
Outline of the QuACK method

In a linear transect kymogram, cytoskeletal structures crossed by the transect produce traces whose temporal dimension reflects the time a structure of interest spent crossing the transect line. With time plotted on the *y* axis of a two-dimensional kymogram (i.e. vertically), the bottom end of a trace corresponds to the arrival of the structure to the transect line (either due to dynamic turnover of its ends or by migration from an out of focus plane), and top end to the moment when the structure left the transect line. Similarly, horizontal trace dimension reflects lateral mobility of the structures crossed by the transect (Fig. 1). Within a range of transect lengths, which has to be determined empirically, distribution of values of longest event duration (longest vertical trace length) should carry information on the structure stability or lifetime (due mainly to assembly/disassembly), while longest lateral displacement (longest horizontal trace length) values should reflect lateral mobility of structures.

To implement this concept, we developed a protocol consisting of several steps, some of them manual, others automated by means of ImageJ macros (Fig. 1). Prior to analysis, an initial selection of raw, microscope-generated video files, which must be all of the same lenght (i.e. timespan and number of frames) is performed based on visual inspection, discarding any obviously incomplete (shorter), out of focus or very noisy recordings, as well as those suffering by visible lateral drift. A sufficient number of biological replicates (individual plants/organs) and technical replicates (repeated recordings from different places of the same organ) should be used. Based on preliminary experiments in *Arabidopsis* seedlings, we recommend analyzing at least 10 recordings from at least five plants, with no more than 2 recordings originating from the same individual, per sample or treatment, and measuring at least 4 transects per recording, as a standard design. This setup was used to generate all the data shown in this paper unless stated otherwise. The number of transects may need to be increased to 6 or 8 to ensure statistical robustness for samples exhibiting only minor differences, or if comparing multiple samples. It is recommended to record at least 20% more video sequences than the above-suggested minimum to ensure that there is enough data left after discarding problematic files. If there is any doubt about the recording quality, it should be kept until the next (contrast enhancement) step, where visual selection is easier to make.

If working with isotropic or non-oriented samples (such as e.g. randomly positioned cotyledons), transects for generating kymograms can be located parallel to the horizontal or vertical edge of the video frame. For anisotropic samples such as e.g. root, hypocotyl or petiole epidermis, recordings must be aligned with a specific axis (e.g. organ axis, or the predominant direction of the cortical microtubule array), positioned either horizontally or vertically, prior to further processing. This can be done using built-in Fiji commands.

Movies are then converted to grayscale and contrast is enhanced by histogram stretching for each frame. This eliminates effects of photobleaching, i.e. gradual loss of fluorescence during recording. A randomly positioned grid is then overlaid over each movie. At this stage, final selection of recordings to be analyzed should be made, discarding videos with excessive amount of random noise (see below for criteria).

Transects of a pre-defined length are then positioned manually along the grid across a well-focused portion of the movie, as equally spaced as possible. (Optical sections from SDCM or VAEM usually have only a part of their area in focus. Thus, automated transect positioning, which would in theory eliminate a source of subjective error, would lead to unacceptable data loss.). Kymograms are then generated across the transects, converted to binary images and skeletonized. All resulting skeletons are manually measured to determine the length of the longest trace in the time (vertical) dimension and the width of the widest trace in the space (horizontal) dimension. Resulting numerical measurements for each condition (or set of experimental parameters) are then processed using a spreadsheet program such as Libre Office Calc or Microsoft Excel and statistically evaluated.

While it is tempting to use statistical approaches such as the *t* test or ANOVA to assess significance of between-group differences, this is not recommended, because long-living events (or large movements) are not completely recorded and thus the data may not fulfill the condition of normal distribution. Other methods, such as the Wilcoxon–Mann–Whitney test or the Kruskal–Wallis test, should thus be used. For the same reason, results should be presented as box plots with median and quartile/range rather than mean values ± SE or SD.

All the ImageJ macros required for performing the above-described procedure are provided, together with detailed step by step instructions, in Additional file 1.

### Validation and optimization on actin data

**Fig. 2:**
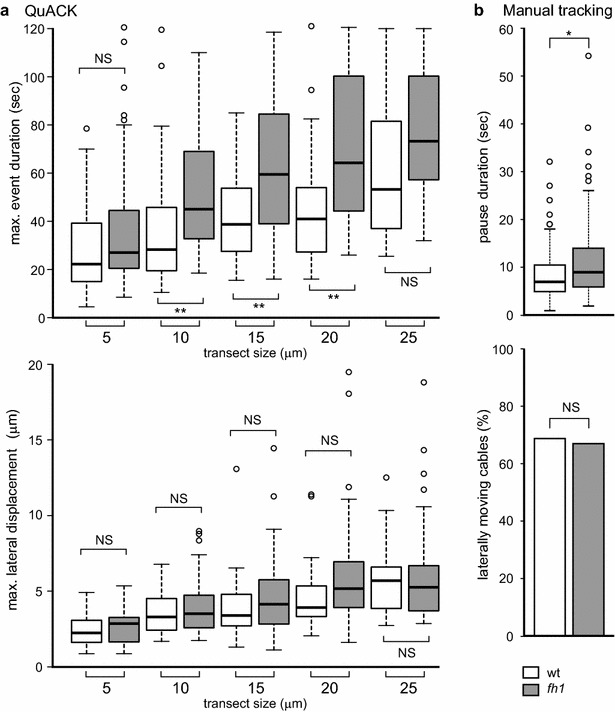
Comparison of QuACK analysis of actin dynamics using varying transect size with manual filament tracking. **a** Actin structure stability (*top*) and lateral mobility (*bottom*) determined by QuACK from SDCM recordings of cotyledon pavement cells of wt and *fh1* seedlings expressing Lifeact-mRFP. **b** Microfilament pause duration (*top*) and fraction of laterally moving actin structures (*bottom*) determined by manual tracking in videos from the same experiment. Statistical significance of differences between wt and *fh1* plants as determined by pairwise Wilcoxon–Mann–Whitney test (with Bonferroni correction in (**a**)) or by Chi square test (in the *bottom panel* of (**b**)) is denoted by *asterisks* (** for *p* *<* 0.01). *NS* not significant (*p* > 0.05)

To test the above-outlined concept, we re-analyzed SDCM recordings of actin dynamics in cotyledon pavement cells of *Arabidopsis* seedlings expressing Lifeact-mRFP, obtained in the course of a previous study [26] where we characterized the effects of a loss of function mutation affecting the formin FH1 (At3g25500). Using manual filament and bundle lifetime measurements, we have shown that mutant *fh1* plants have more stable actin structures in cotyledon pavement cells than wild type (wt) plants. We have re-measured the raw data previously used to generate Fig. 6 of [26] using the QuACK protocol, varying the transect length to obtain an estimate of the robustness of the assay towards this parameter (Fig. 2a). Consistent with results of manual filament tracking, which showed increased pause duration in the mutant plants, the results of QuACK analysis document a significant increase in actin structure lifetime (i.e. stability) in *fh1* plants compared to the wt for all but the shortest transect length examined. Lateral mobility of actin structures, estimated either using QuACK or by manual tracking, did not show significant differences (Fig. 2a, b). Upon visual inspection of the source SDCM recordings and kymograms it was rather obvious that the difference between mutant and wt plants can be attributed to the presence of long-living, stable actin bundles in the former (see also [26]). Since these bundles were relatively sparse, short transects led to a substantial fraction of mutant kymograms missing them. To detect the influence of such comparably rare events, transect size should be chosen as long as possible, i.e. the longest that can be easily accommodated into the well-focused part of all movies under study. We found such an approach generally appropriate also for other analyses involving the rapidly moving cortical microfilaments (see below).

Besides of missing relatively rare events (such as the stable actin bundles in the *fh1* mutant), there is a theoretical possibility that frequent presence of stable structures might result in all or nearly all kymograms containing events that reach the maximum available value (i.e. the whole duration of the recording in case of lifetime values). While this was never a problem with actin dynamics, we had to address this issue when applying QuACK to microtubules (see below).

**Fig. 3:**
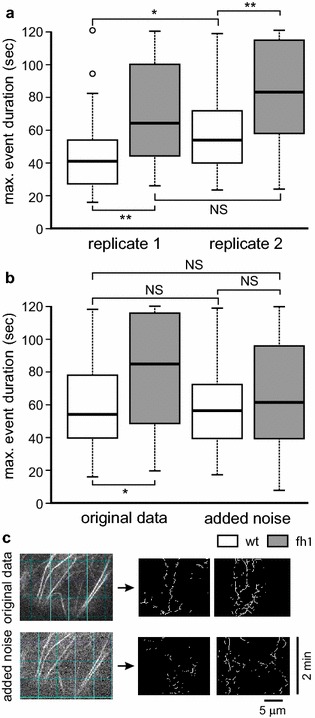
Reproducibility of QuACK results. **a** Actin structure stability in cotyledon pavement cells of wt and *fh1* seedlings expressing Lifeact-mRFP, determined from SDCM recordings from two independent experiments involving separate cultivations using QuACK with transect length of 20 μm. While the actual values measured for each genotype vary between experiments, the presence and direction of inter-genotype differences is reproducible. **b** Actin structure stability estimated as in (**a**) from a set videos obtained in Experiment 1, analyzed either in their original state or after controlled introduction of noise using the ImageJ “Process–Noise–Add specified noise” command with standard deviation value set at 100. *Asterisks* in (**a**) and (**b**) denote statistical significance at *p* *<* 0.05 (*) or *p* *<* 0.01 (**) determined by pairwise Wilcoxon–Mann–Whitney test with Bonferroni correction. *NS* not significant (*p* > 0.05). **c** Sample frames from an original video (*top*) and the same frame after noise introduction (*bottom*), together with representative kymograms. Grid = 5 μm. Only recordings comparable with the original (*top*) video but not with the noisy (*bottom*) one should be considered for analysis

Experiment to experiment reproducibility is a serious concern for any quantitative method. While we did observe some variability in absolute measured values of actin structure lifetime between measurements on independent plant cultures (which have differed by several hours of age and could have been influenced e.g. by a minor variation of growth chamber temperature), the direction and relative size of differences between wt and *fh1* actin structure lifetime values was well reproducible (Fig. 3a). Nevertheless, we have to stress the importance of only directly comparing plants that have been sown, grown and recorded simultaneously. We also found that a good signal to noise ratio is required for reliable QuACK measurement. For instance, VAEM recordings of GFP-FABD-tagged cortical actin, which have been previously successfully, though very laboriously, evaluated by manual methods [26], did not produce reliably measurable kymograms in our hands.

To further examine the effects of noise on QuACK analysis results, we compared actin structure lifetime obtained from a particular wt and *fh1* dataset using the standard protocol with results derived from the same videos previously subjected to controlled introduction of random noise. On these noisy data, the difference between mutant and wt actin dynamics appears to be diminishing (Fig. 3b), obviously due to fragmentation of long kymogram traces (Fig. 3c), which were mainly responsible for the increased lifetime in the mutant. Thus, to avoid artifacts due to trace fragmentation, video recordings with a similar level of noise should be compared, and very noisy files should be discarded prior to kymogram construction. The level of noise can be best assessed visually after the contrast adjustment and grid insertion step (Fig. 3c). If comparison between videos of varying noise levels cannot be avoided, interpretation should reflect the tendency towards loss of long traces in noisy data.

### Validation and optimization on microtubule data

Microtubules in plant cells are generally easier to visualize with an excellent signal to noise ratio, and their dynamics, slower than in case of microfilaments, is also relatively easy to quantify. Nevertheless, the general limitations of conventional methods, as outlined in the Introduction, apply to them as well. We thus examined the applicability of the QuACK approach to microtubules using existing VAEM recordings of cortical microtubule dynamics in cotyledon pavement cells of wt and *fh1* mutant *Arabidopsis* seedlings expressing GFP-MAP4, produced for our recently published study [26]. Individual microtubule end tracking has previously revealed an increased microtubule end turnover and decreased lateral microtubule end movement in the mutant compared to the wt, while an overall increase of microtubule mobility in the mutant was determined by image to image correlation measurements [26].

**Fig. 4:**
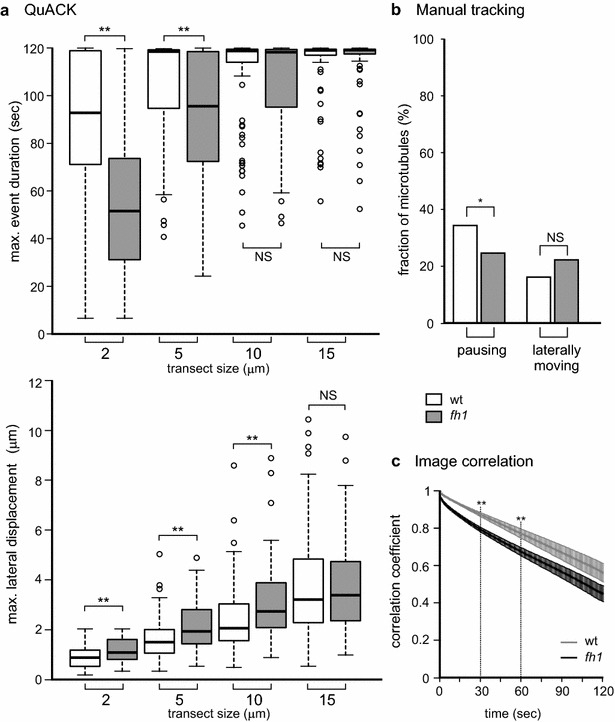
Comparison of QuACK analysis of microtubule dynamics using varying transect size with other methods. **a** Microtubule stability (*top*) and lateral mobility (*bottom*) determined by QuACK from VAEM recordings of cotyledon pavement cells of wt and *fh1* seedlings expressing GFP-MAP4. Eight transects per movie were measured. **b** Fraction of microtubules at pause and exhibiting lateral movements determined by manual tracking in videos from the same experiment. Statistical significance of differences between wt and *fh1* plants as determined by pairwise Wilcoxon–Mann–Whitney test with Bonferroni correction in (**a**) and by Chi square test in (**b**) is denoted by *asterisks* (** for *p* *<* 0.01, * for *p* *<* 0.05). *NS* not significant (*p* > 0.05). **c** Image to image correlation curves from the same data (modified from [26]). *Asterisks* denote statistically significant difference between wt and *fh1* plants at time points denoted by broken lines (Tukey HSD, ** for *p* *<* 0.01)

Analysis of the same data by QuACK with varying transect length (Fig. 4a) revealed an overall high frequency of stable microtubules, leading to a loss of resolution with longer transects that nearly always contained events whose duration exceeded the length of the recording. Reducing the transect length resulted in detection of a major and highly significant difference in microtubule lifetime, confirming the previously observed increased microtubule turnover in *fh1* mutants. Lateral mobility of microtubules was also found to be higher by this approach over a range of transect lengths. Individual filament tracking confirmed a decreased fraction of pausing microtubules in the mutant but did not detect significant differences in lateral motility (assessed by following points randomly located along microtubules rather than ends). However, a non-significant trend towards greater lateral motility was observed in the mutant (Fig. 4b), suggesting that inclusion of more data (hundreds of filaments) might show an increase in lateral microtubule movements. Image-to-image correlation analysis indicated a highly significant increase in overall microtubule dynamics in *fh1* plants (Fig. 4c).

**Fig. 5:**
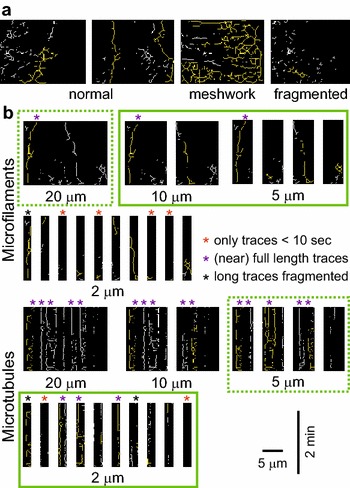
Appearance of skeletonized kymograms—considerations for data quality control and transect size selection. **a** Representative skeletonized kymograms obtained from SDCM recordings from cotyledon epidermis of wt seedlings expressing Lifeact-mRFP using a 20 μm transect. With good quality video recordings, most kymograms should contain one or a few distinct long traces (*left*), with only a fraction possibly showing a meshwork or fragmented pattern (*right*). Too many meshwork kymograms result in most lifetime and lateral motility values close or equal to the maximum, preventing meaningful interpretation, while fragmented kymograms are indicative of noisy and thus possibly unreliable primary data. **b** Skeletonized kymograms obtained from the same place within a representative SDCM recording from *fh1* cotyledon epidermis expressing Lifeact-mRFP (*top*) or from a VAEM recording of wt cotyledon epidermis expressing GFP-MAP4 (*bottom*) using the indicated transect sizes. *Green frames* indicates a recommended range of transect sizes for capturing both rare and frequent events (*dotted line* means sub-optimal, but possibly still usable, settings). While these examples illustrate lifetime measurements, analogous considerations also apply for quantification of lateral motility. Longest traces in the time dimension are shown in *yellow*, *asterisks* denote specific problems or features (see key in the figure)

Thus, QuACK can be used to analyze the dynamics of both actin and microtubules. We, however, recommend performing preliminary (calibration) analyses of several recordings for any given type of data (and preferentially for multiple experimental variants studied) before embarking on any large-scale study to verify data quality (Fig. 5a) and to optimize transect size for maximum sensitivity. Transect size should be adjusted to utilize the whole span of available event duration (or lateral mobility) values. Settings that result in either very low or very high (full axis length) values in two-thirds of skeletonized kymograms or more, as well as those resulting in fragmentation of nearly all long traces, should be avoided; at best, at least a half of all kymograms should produce non-extreme values (Fig. 5b).

We have next employed QuACK to address two model questions, namely (i) how do various fluorophores affect the dynamics of microfilaments tagged by constructs utilizing the same actin-binding moiety, and (ii) how anisotropic are the lifetime and lateral mobility parameters of cortical microfilaments and microtubules in differentiated elongated plant cells.

### Case study I: effects of fluorescent protein markers on actin dynamics

**Fig. 6:**
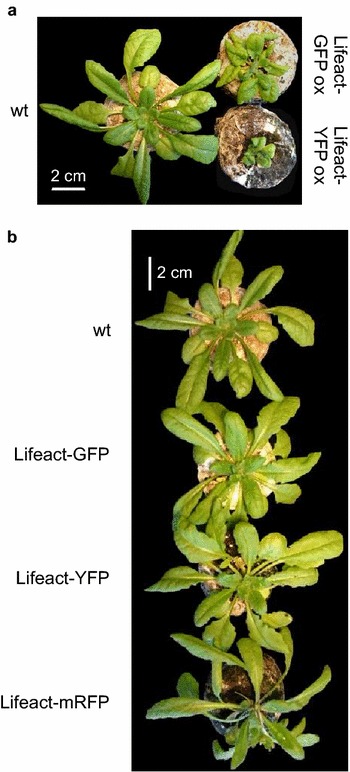
Phenotypic consequences of Lifeact derivatives expression in 4 weeks old *A. thaliana* plants. **a** A comparison between a non-transgenic (wt) rosette and two transformants exhibiting very high levels of Lifeact-GFP and Lifeact-YFP expression as observed by conventional fluorescence microscopy (overexpression–ox lines). **b** Plants exhibiting moderate levels of marker expression, similar to those used for most subsequent analyses

We generated transgenic *Arabidopsis* lines expressing Lifeact fused to either GFP or YFP under the control of the UBQ10 (ubiquitin10) promoter. The promoter and fluorophores were chosen to avoid marker silencing during intended future use of these markers in backgrounds carrying various T-DNA alleles [39] and to minimize interference with actin organization. While strong overexpression of both variants led to plant growth perturbations caused probably by high Lifeact level itself, transformants with moderate levels of fluorescence resembled wt plants in all macroscopic aspects throughout their life cycle (Fig. 6). Lifeact dose-dependent reduction of actin dynamics, known to occur in plants free of obvious phenotypic changes [11], was minimized by selection of transformants showing comparable and rather low fluorescence levels upon visual inspection; progeny of such plants was used for further experiments. Pilot fluorescent microscopy observations have been performed with at least 10 independently transformed lines, initial SDCM observations with progeny of at least 2 transformants exhibiting visually similar actin behavior. Below we report results obtained on a mixture of seedlings originating from two typical, representative transformed lines for each marker. A previously established line expressing Lifeact-mRFP under the 35S promoter [40] was included for comparison.

**Fig. 7:**
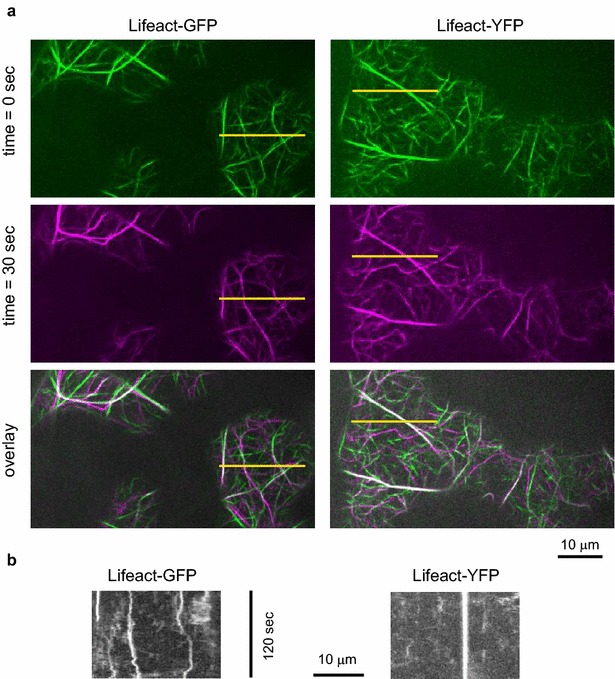
Actin dynamics in seedling cotyledons, visualized using Lifeact-GFP and Lifeact-YFP markers. **a** SDCM images from two time points and their overlay, showing filaments with altered localization (*green* and *magenta*), as well as those which remained static (*white*). Note stable bundles in the Lifeact-YFP expressing line. **b** Kymograms generated from transects marked by *yellow lines* in (**a**)

**Fig. 8:**
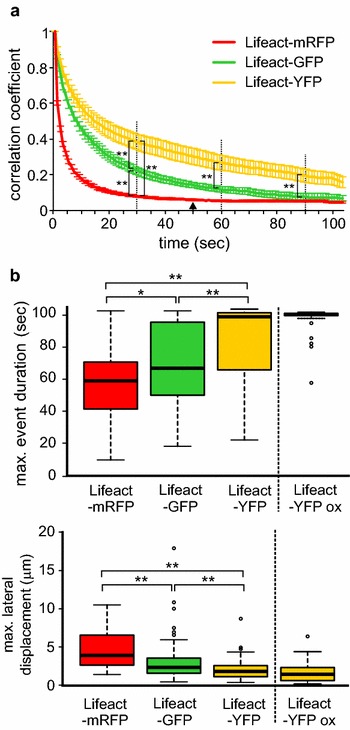
Effects of various fluorophores on actin dynamics as detected using Lifeact fusion proteins. Data from cotyledons of wt seedlings, recorded for 105 s. **a** Image to image correlation curves for representative transformants carrying the indicated Lifeact fusion proteins. Note that for Lifeact-mRFP the correlation value becomes time-independent, and thus the assay non-informative, at approximately 50 s (*arrow*). *Asterisks* denote statistical significance of the indicated differences at time points denoted by broken lines (Tukey HSD, ** for *p* *<* 0.01). **b** QuACK results from the same data, with a massively overexpressing Lifeact-YFP line (Lifeact-YFP ox) added for comparison but not included in statistical calculations. Presence of differences was confirmed by the Kruskal–Wallis test with *p* *<* 0.01. Statistical significance of differences between the indicated samples determined by Wilcoxon–Mann–Whitney test with the Bonferroni correction is denoted by * for *p* *<* 0.05, ** for *p* *<* 0.01. Transect size was 20 μm. Between 50 and 70 transects per sample were measured

Both Lifeact-GFP and Lifeact-YFP labeled actin populations including very fine filaments and exhibited higher stability of fluorescence (i.e. reduced bleaching) compared to the mRFP variant, allowing better visualization of actin network over time. Preliminary SDCM observations, however, revealed noticeable differences in actin structure and dynamics between plants carrying the Lifeact-GFP and Lifeact-YFP markers, with the later exhibiting apparently reduced lateral mobility and increased lifetime of actin filaments and bundles (Fig. 7; Additional files 2 and 3). Since we managed to obtain high quality SDCM video recordings suitable for image-to-image correlation analysis, we used this method to quantify overall actin dynamics in plants carrying Lifeact derivatives with different fluorophores (Fig. 8a). While Lifeact-GFP and Lifeact-YFP produced reliable data throughout the observation time, Lifeact-mRFP not only exhibited fast actin reorganization, but also bleached rapidly, leading to decrease in signal to noise ratio and non-informative results after first 50 s of recording. Nevertheless, before the correlation values became time-independent, Lifeact-mRFP turned out to be the most dynamic of all three variants tested. Surprisingly, however, a highly significant difference in actin dynamics was found also between Lifeact-GFP and Lifeact-YFP expressed under the same promoter, with the later consistently decreasing actin mobility.

We used the same data for quantification of actin dynamics using QuACK, with inclusion of an additional Lifeact-YFP-expressing line which exhibited very high fluorescence intensity and some reduction in growth and morphological abnormalities (the Lifeact-YFP overexpression, or Lifeact-YFP ox, line). The qualitative differences previously detected by the image to image correlation technique were confirmed, and an even more obvious decrease in actin dynamics was apparent in the Lifeact-YFP ox line (Fig. 8b). In addition, QuACK analysis revealed that the observed differences are due to significant differences both in actin structures lifetime and their lateral mobility.

### Case study II: anisotropy of cytoskeletal dynamics in elongated cells

Spatial anisotropy of cytoskeletal organization in elongating or elongated cells is a phenomenon is well-documented especially for microtubules [41, 42], but reported also for actin [43]; however, much less is known about the anisotropy of cytoskeletal dynamics in such cells.

**Fig. 9:**
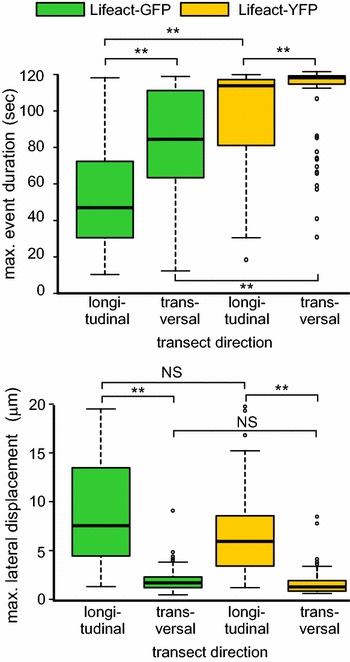
Actin bundle turnover and lateral mobility is anisotropic in elongated cells. SDCM data from 1st or 2nd true leaf petioles of two weeks old wt seedlings expressing Lifeact-GFP and Lifeact-YFP. Actin structure stability (*top*) and lateral mobility (*bottom*) were determined by QuACK with 20 μm transects positioned in the indicated direction. Presence of differences was confirmed by the Kruskal–Wallis test with *p* *<* 0.01. Statistical significance of differences between the indicated samples determined by Wilcoxon–Mann–Whitney test with the Bonferroni correction is denoted by ** for *p* *<* 0.01; *NS* not significant (*p* > 0.05)

We used QuACK analysis of SDCM recordings, with transects oriented either alongside cell and organ axis (i.e. in the longitudinal direction) or perpendicular to it (i.e. in the transversal direction), to characterize microfilament behavior in elongated epidermal cells of true leaf petioles expressing Lifeact-GFP or Lifeact-YFP. Like in cotyledon pavement cells (Fig. 8b), actin filaments reorganized faster in the Lifeact-GFP transformants compared to Lifeact-YFP ones, regardless of transect direction. However, unlike in the case of cotyledon pavement cells, no significant difference between markers in lateral mobility was observed in the petiole epidermis, indicating cell type- or tissue-specific differences in this aspect of microfilament dynamics (Fig. 9). Remarkably, actin structures crossed by transects perpendicular to the organ axis (and thus enriched in filaments or bundles oriented parallel to this axis) exhibited significantly increased lifetime and decreased lateral mobility compared to those crossed by transects parallel to the organ axis for both fluorophores (Fig. 9). Thus, elongated cells exhibit marked anisotropy in both actin filament or bundle lifetime and lateral mobility depending on their orientation.

**Fig. 10:**
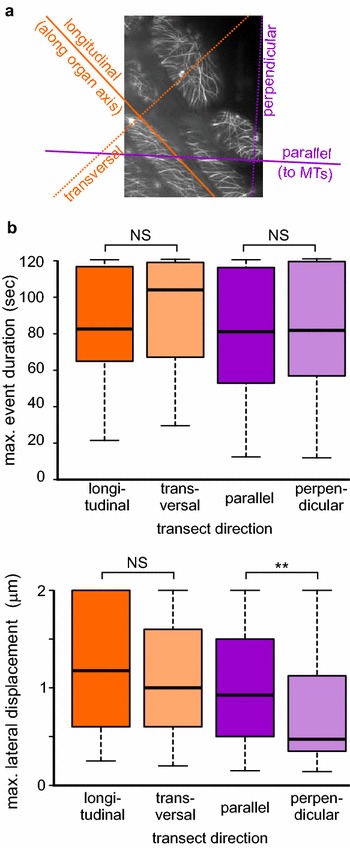
Lateral mobility of microtubules, but not microtubule end dynamics, is anisotropic in elongated cells. Data from VAEM recordings of 20–30 hypocotyl epidermal cells from 3 wt seedlings expressing GFP-MAP4. Two 2 μm transects per cell and direction were measured. **a** Definition of transect directions used. **b** Microtubule stability (*top*) and lateral mobility (*bottom*) measured by QuACK on transects following the directions shown in (**a**). Statistical significance of differences between the indicated directions determined by Wilcoxon–Mann–Whitney test is denoted by *asterisks* (** for *p* *<* 0.01). *NS* not significant (*p* > 0.05)

To our surprise, we did not detect any significant differences in either lifetime or lateral mobility when analyzing VAEM recordings of GFP-MAP4-tagged cortical microtubules in the epidermis of light-grown hypocotyls by QuACK, comparing longitudinal transects to transversal ones (Fig. 10), despite the microtubule organization exhibiting very obvious anisotropy, with prominent spiral arrays oriented obliquely with respect to the cell and organ axis. However, QuACK analysis with transects oriented parallel to these arrays (rather than the organ or cell axis) compared to those perpendicular to the predominant array direction revealed a significantly lower lateral mobility of microtubules crossed by the perpendicular axis, i.e. in a subpopulation enriched in microtubules participating in the construction of the array.

## Discussion

Here we present a new method, QuACK (Quantitative Analysis of Cytoskeletal Kymograms) for evaluating the in vivo dynamics of plant cortical cytoskeleton from SDCM or VAEM video recordings obtained from cells tagged by expression of actin- or microtubule-specific fluorescent protein markers. Our approach is based on quantitative analysis of kymograms, projections of the video recordings to two dimensions with a transect across the image field providing the *x* dimension and time contributing the *y* dimension. Kymograms are alternatively termed “kymographs” in the literature (e.g. [37, 44]), albeit this word probably ought to be reserved to hardware or software devices producing these graphical representations of motion, at least per analogy with similar terms such as “telegram” versus “telegraph” [45]. While kymogram-based techniques have been introduced into quantitative cytoskeletal dynamics studies previously, they were usually used to follow the movement of individual filaments, bundles or filament ends along linear or curved trajectories with subsequent analyses of population behavior (e.g. [37, 44, 46]) rather than for capturing parameters reflecting the overall dynamics of the actin or microtubule networks. Moreover, these methods are often based on sophisticated theoretical background that may make them less than user-friendly from the point of view of experimental biologists. This is also the case of the (to our knowledge only) plant study employing kymogram analysis to study global microtubule dynamics [47].

QuACK presents several advantages over commonly used techniques for estimating cytoskeletal dynamics, such as manual tracing of individual filaments or their ends (e.g. [3, 23, 25, 26, 33, 34]) or automated image to image correlation [35, 36]. It is faster than manual tracing, relatively simple and intuitive. Unlike the correlation method, it independently estimates the contribution of dynamic filament end turnover (reflected in the maximum event duration parameter) and lateral mobility (expressed as the maximum lateral displacement parameter) to overall dynamics of the structures under study. QuACK analysis can be performed using exclusively free software on any commonly used operation system, since both ImageJ/Fiji and freely available spreadsheet programs exist in versions compatible with Microsoft Windows, Linux and Mac-OS. While the method involves some manual steps that introduce a theoretical possibility of observer bias, we found this not to present a practical problem during testing (as even experienced observers could not predict the appearance of the skeletonized kymogram from the source video). Results of our method appear to be somewhat sensitive to the amount of noise in input video data. However, we provide suggestions on post hoc qualitative evaluation of input data based on the frequency of fragmented traces and meshwork patterns.

To validate our approach, we re-examined raw video data from our published study examining the effects of functional impairment of the *Arabidopsis* FH1 formin (encoded by the At3g25500 locus) on cortical actin and microtubule dynamics [26]. Using static image analysis and manual tracking of filaments labeled with several different marker proteins, we have previously shown that *fh1* mutants exhibit increased microfilament bundling and thus increased actin structure lifetime both in rhizodermis and in cotyledon pavement cells [25, 26], possibly as a consequence of weakening the trans-plasmalemma attachment of cortical microfilaments to the cell wall [48] that may allow for easier lateral movement and thus bundling of individual microfilaments [49]. QuACK analyses convincingly confirmed increased lifetime of actin structures in *fh1* mutants. However, lateral movement of recorded actin structures did not significantly differ between the mutant and the wt, most likely because increased lateral mobility of individual microfilaments was compensated for by decreased mobility of the frequently occurring thick bundles in the mutant. Indeed, such extremely stable bundles are frequently found in mutant plants [25, 26]. At present, QuACK does not distinguish between thick and thin bundles or even single filaments. While development in such a direction is conceivable, utilizing, e.g., thresholding functions of ImageJ, it is beyond the scope of the present work.

Unlike microfilaments, microtubules are generally more dynamic in *fh1* mutants than in wt plants, as determined by individual filament end tracking, which showed increased dynamic instability of microtubule ends [25, 26], and by the image to image correlation method that documented an overall increase in microtubule rearrangements [26]. Using QuACK, we have demonstrated both a decrease in microtubule lifetime and an increase in overall lateral mobility in the *fh1* mutants. The later is somewhat surprising, since decreased frequency of microtubule end translocations (“end waving”) was found in the mutant. Since QuACK cannot distinguish between lateral movements of whole microtubules and their ends, our observations suggest that the later may represent only a minor fraction of overall microtubule mobility. This is also consistent with the change in microtubule end behavior being one of the very few aspects of the *fh1* mutant phenotype that could not be replicated by pharmacological inhibition of formin activity [26], and with the noticeable, though not statistically significant, trend towards increased lateral microtubule mobility detected by individual microtubule tracking in the present study.

After validating and optimizing the QuACK method on the formin mutant data, we employed this approach to characterize microfilament dynamics in *A.* *thaliana* lines expressing the actin marker Lifeact fused to various fluorophores, expressed under the control of the UBQ10 promoter, previously proposed as a promoter of choice for live cytoskeletal labeling due to its ability to achieve relatively low expression levels, minimizing thus overexpression artifacts [9]. While transgenes driven by the 35S promoter often exhibit silencing when crossed into the genetic backgrounds associated with commonly used T-DNA lines, including the SALFK collection [39], no such silencing occurred in our lines after crosses into several SALK-derived lines which previously exhibited silencing of 35S promoter constructs past the early seedling stage [26]. Thus, actin can be now followed in all developmental stages of *Arabidopsis* mutants.

We found surprising differences in both microfilament lifetime and lateral mobility depending on the fluorophore used, even at comparable marker expression levels. Lifeact-YFP-expressing plants had less dynamic actin structures compared to those carrying Lifeact-GFP, while microfilaments labeled by Lifeact-mRFP were more dynamic than Lifeact-GFP-tagged ones. Fluorophore-dependent differences in actin organization have been reported for fimbrin- based markers, with mOrange labeling aggregated rather than filamentous structures compared to GFP-, YFP- or CFP-based tags [9]. While mOrange is a derivative of dsRed, a protein that, unlike GFP, must oligomerize in order to become fluorescent, it is a descendant of a non-dimerizing mutant [50]; the reported effects are thus unlikely to be due solely to fluorophore dimerization or oligomerization. However, differences in fluorophore sequence (and thus structure) might affect the tagged proteins´ interactions with microfilaments or with proteins modulating actin organization and dynamics, leading to selective tagging of distinct microfilament structures or to changes in tagged structure behavior. In case of Lifeact, a recent study demonstrated that neither the position nor the identity of the fluorophore (mEGFP or mCherry) affected its affinity to actin in vitro. Interestingly, at low concentrations, Lifeact-mCherry even promoted actin nucleation and filament elongation, while at high doses it exhibited a clear inhibitory effect [51]. The latter is also consistent with inhibitory effects of high levels of Lifeact expression on growth observed by others [9] and in our present study. However, the differences between Lifeact-GFP and Lifeact-YFP behavior are rather surprising, given the 98% sequence identity between these proteins, which differ only in several amino acids located inside the folded tertiary structure [52]. Major changes in protein surface properties are thus unlikely, albeit subtle changes in conformation may affect protein–protein interactions. Nevertheless, YFP fusions of the prokaryotic actin homolog MreB were reported to exhibit distinctly filamentous organization not reproduced by homologous GFP-tagged constructs in another bacterial species [53], hinting at possible hitherto overlooked effects of subtle fluorophore differences. In any case, our data show that there can never be too much caution when interpreting results obtained using fluorescent protein tags expression.

We have next employed QuACK for initial characterization of spatial anisotropy of cytoskeletal dynamics in two markedly polar cell types, namely in differentiated, elongated epidermal cells of true leaf petioles (where actin was observed) and light-grown hypocotyls (where we followed microtubule behavior).

While much attention has been devoted to the signaling and regulatory aspects of actin rearrangements [54], very few studies focus on the anisotropy of microfilament organization and dynamics, usually in the context of pathological situations [55, 56], cell division [57] or tip growth [58] rather than during or following diffuse anisotropic cell expansion. Our analyses in mature elongated epidermal cells document decreased turnover and lateral mobility of actin filaments or bundles parallel to the longitudinal cell axis compared to those crossing this axis. This supports the established model (derived in part from work in *Chara* and in tip-growing cells) where longitudinal actin bundles provide “tracks” for cytoplasmic streaming, which can be expected to be rather stable compared to the rest of the highly dynamic actin meshwork [59–61]. Actin organization and dynamics is also known to be intimately interlinked with that of microtubules (e.g. [30, 62]); it thus remains to be clarified what is the role of the microtubule cytoskeleton in controlling the anisotropic behavior of microfilaments.

Compared to microfilaments, the role of cortical microtubules in polarized diffuse cell growth is well characterized [41, 42]. In longitudinally expanding cells, microtubules undergo constant dynamic rearrangements [22] resulting in gradual rotation of highly organized cortical arrays, whose orientation correlates with that of nascent cellulose microfibril deposition [63]. Cortical microtubules with reduced lateral mobility tend to form arrays perpendicular to the direction of maximal growth in expanding shoot apical meristem cells [64]. Our observations in fully expanded *Arabidopsis* hypocotyl cells suggest a reciprocal conclusion—namely that those microtubules which are collinear with the array tend to be less mobile. The cortical array can therefore be understood as an “attractor” stabilizing the position of microtubules once they become part of it.

Indirect evidence suggests that the stability of cortical microtubules may reflect their orientation, since in growing maize coleoptiles transversal microtubules participating in the cortical array are enriched in de-tyrosinylated tubulin that inhibits microtubule turnover [65]. While we did not observe increased lifetime of microtubules aligned to the cortical array, predicted by this hypothesis, we did find that these microtubules are less laterally mobile. It is, of course, possible that observations from growing maize coleoptiles cannot be generalized to fully expanded epidermal cells of *Arabidopsis* hypocotyls (and vice versa). The differences in lateral microtubule mobility observed in our experiments might still reflect different post-translational modifications of aligned *versus* non-aligned microtubules, resulting in altered affinity towards microtubule-associated proteins that control lateral mobility, possibly via linking microtubules to the cell wall matrix through transmembrane proteins such as e.g. certain formins [48, 66] or components of the cell wall biosynthesis machinery [67].

In any case, we hope that QuACK can be used as a versatile tool in future studies aimed at closer characterization of cytoskeletal dynamics anisotropy in a variety of cell types, developmental situations and environmental contexts.

## Conclusions

We provide a simple, freeware-based, operational system-independent method for estimating the lifetime and lateral mobility of fluorescent protein-tagged cytoskeletal structures in plant cells. The technique, which is suitable for analyzing the dynamics of both actin and microtubules from SDCM or VAEM recordings, was validated by confirming the previously documented increase in microfilament assembly stability and microtubule turnover in *Arabidopsis fh1* mutants and then applied to characterize the unexpected effects of various fluorescent marker fusions on the dynamics of the actin marker Lifeact, as well as to obtain initial insights into the anisotropy of the turnover and lateral mobility of cortical actin and microtubules in elongated plant cells.

## Methods

### Plants

*A.* *thaliana* ecotype Columbia lines carrying the Lifeact-mRFP [40] and GFP- MAP4 [14] markers in wt or *fh1*-*1* mutant background have been described previously [25, 26]. Lifeact-GFP and Lifeact-YFP marker lines were prepared using following constructs: Lifeact in pDONR207 [40] was transferred to the pUBC-GFP-dest and pUBC-YFP-dest vectors [68] by the LR Clonase II (Invitrogen). Both constructs were then transformed into *A.* *thaliana* Col-0 plants using the floral dip method and transformants selected using BASTA. Seeds produced by T2 generation plants have been used to grow seedlings for the experiments reported here.

For propagation and transformation, plants were grown in soil or peat pellets (Jiffy). Seedlings for imaging were grown in vitro at 22 °C with a 16 h light/8 h dark cycle on vertical Murashige and Skoog (MS) plates from surface-sterilized seeds stratified by several days of post-imbibition storage at 4 °C to synchronize germination. 5 day old seedlings were observed unless stated otherwise.

### Image data acquisition

Image series (videos) were recorded for 2 min unless stated otherwise from seedlings mounted in water on chambered slides as described previously [3, 25, 26]. SDCM recordings were performed using an inverted spinning disc confocal microscope (Yokogawa CSU-X1 on a Nikon Ti-E platform, laser box Agilent MLC400, camera Andor Ixon) with plan apochromat × 100 oil (NA = 1.45) lens, laser lines set at 488 and 561 nm, and image interval 1 s. VAEM recordings were generated using the Leica AF6000 LX fluorescence platform with integrated TIRF module and a Leica DFC350FXR2 digital camera, with plan apochromat × 100 oil (NA = 1.46) lens, 400 nm peak excitation, and 210 ms exposure time, allowing for image interval 0.5 s.

### Image data analysis

The Fiji distribution of ImageJ [27] was used for all image analysis steps except frame-to-frame autocorrelation measurements. Microfilament pause duration and fraction of pausing microtubules was quantified by visual end tracking (aided by manual labeling of tracked structures in ImageJ) as described previously [26]. Lateral motility of microfilament structures was assessed after prior uniform contrast enhancement of video recordings by scoring the presence or absence of movement for randomly selected points located within a structure (using the point tool in ImageJ) over the span of 10 frames. Lateral motility of microtubules was determined in an analogous manner, following the fate of selected points over the span of 2 min. In all manual analyses, at least 120 filament ends or points in at least 12 cells from at least 7 plants per genotype were evaluated.

A summary of the QuACK method is described in Results and a detailed protocol provided in Additional file 1. Before QuACK measurements in anisotropic samples, a straight marker line was manually placed along a visually detected landmark, i.e. the organ axis or several parallel thick bundles of the cortical microtubule array. In the later case, marker line direction was chosen to minimize intersections with prominent cortical microtubules or bundles. Subsequently, the movie frame was rotated to align the marker line with the horizontal axis (for detailed instructions see Additional file 1).

To determine overall microtubule or actin dynamics by the image-to-image correlation method, correlation coefficients of pairs of video frames separated by varying intervals were calculated according to [35], using at least 10 movies from at least five plants per sample.

### Statistics

Online utilities [69–71] or a freely available MS Excel template [72] have been used for statistical evaluation using the Kruskal–Wallis, Wilcoxon–Mann–Whitney and Chi square tests. The various tools were used alternately after verification that they provide identical results on a subset of data. Box plots were generated using the BoxPlotR tool [73].

## Additional files

**Additional file 1.** Detailed protocol and ImageJ macros for performing QuACK measurements. The .cab archive file (which can be unpacked using free programs such as e.g. Cabextract or Keka on non-Windows systems, or using free web utilities such as the online version of the B1 archiver at http://online.b1.org/online) contains three ImageJ/Fiji macros (.ijm) and a documentation (.pdf) file with detailed step-by-step setup and application instructions.

**Additional file 2.** A representative SDCM video recording of actin dynamics in cotyledon pavement cells of an *A.* *thaliana* seedling expressing GFP-Lifeact. Scale bar = 10 μm (.avi video file).

**Additional file 3.** A representative SDCM video recording of actin dynamics in cotyledon pavement cells of an *A. thaliana* seedling expressing YFP-Lifeact. Scale bar = 10 μm (.avi video file).

## Abbreviations

FH1: Formin homolog 1
GFP: Green fluorescent protein
MAP4: Microtubule-associated protein 4
mRFP: Monomeric red fluorescent protein
ox: Overexpression
QuACK: Quantitative analysis of cytoskeletal kymograms
SDCM: Spinning disc confocal microscopy
UBQ10: Ubiquitin 10
VAEM: Variable angle epifluorescence microscopy
wt: Wild type
YFP: Yellow fluorescent protein
